# Inflammatory Proteomic Heterogeneity Beyond Glycemia Status in Severe Obesity

**DOI:** 10.3390/ijms27094152

**Published:** 2026-05-06

**Authors:** Melissa M. Milito, Mattia Chiesa, Alice Mallia, Giulia G. Papaianni, Julia T. Regalado, Claudio Tiribelli, Deborah Bonazza, Natalia Rosso, Silvia Palmisano, Cristina Banfi, Pablo J. Giraudi

**Affiliations:** 1 Metabolic Liver Disease Unit, Fondazione Italiana Fegato, 34012 Trieste, Italy; melissa.milito@fegato.it (M.M.M.); ctliver@fegato.it (C.T.);; 2 Department of Life Sciences, University of Trieste, 34127 Trieste, Italy; 3 Bioinformatics and Artificial Intelligence Facility, Centro Cardiologico Monzino IRCCS, 20138 Milan, Italy; mattia.chiesa@cardiologicomonzino.it; 4 Unit of Functional Proteomics, Metabolomics, and Network Analysis, Centro Cardiologico Monzino IRCCS, 20138 Milan, Italy; alice.mallia@cardiologicomonzino.it (A.M.);; 5 Department of Science and Technology, Philippine Council for Health Research and Development, Bicutan, Taguig 1631, Philippines; 6 Surgical Pathology Unit, Cattinara Hospital, Azienda Sanitaria Universitaria Giuliano Isontina, 34149 Trieste, Italy; 7 Surgical Clinic Division, Cattinara Hospital, Azienda Sanitaria Universitaria Giuliano Isontina, 34149 Trieste, Italy; 8 Department of Medical, Surgical and Health Sciences, University of Trieste, 34149 Trieste, Italy

**Keywords:** type 2 diabetes, inflammatory proteomics, topological data analysis, low-grade inflammation, immune phenotypes

## Abstract

Chronic low-grade inflammation is a key feature of obesity-associated dysglycemia, yet substantial heterogeneity exists in inflammatory responses among individuals with normoglycemia, prediabetes, and type 2 diabetes mellitus (T2DM). Whether circulating inflammatory protein profiles define distinct patient phenotypes beyond conventional glycemic classification remains incompletely understood. In this cross-sectional analysis of 142 individuals with severe obesity, plasma inflammatory proteins were quantified using Olink proximity extension assay technology. Subjects were stratified by glycemic status (noDM, normoglycemia; PreDM, prediabetes and T2DM) while maintaining comparable distributions of metabolic dysfunction-associated steatotic liver disease. Differential expression analyses were performed across glycemic groups, and unsupervised topological data analysis (TDA) was applied to identify inflammatory protein-based patient subgroups. Several inflammatory proteins were significantly upregulated in T2DM and PreDM compared with noDM, with interleukin-8 (IL-8), Fms-relatedlike tyrosine kinase 3 ligand (Flt3L), and CUB domain containing protein (CDCP1) showing the largest significant differences. NPX distributions of these proteins exhibited gradual increases across glycemic stages with substantial inter-individual variability. TDA identified seven clusters defined by distinct inflammatory protein signatures. One cluster was enriched for individuals with T2DM and characterized by coordinated upregulation of IL-8, Flt3L, CDCP1, and additional immune- and cytokine-related proteins, whereas other clusters displayed alternative inflammatory profiles that were not explained by glycemic status alone. Inflammatory proteomic profiling in severe obesity reveals both glycemia-associated protein changes and distinct inflammatory phenotypes that transcend conventional clinical classification. Integration of differential expression analysis with TDA highlights heterogeneity in inflammatory states, supporting a hypothesis-generating framework for future studies aimed at validating these proteomic patterns and clarifying their longitudinal relevance in obesity-related dysglycemia.

## 1. Introduction

Obesity represents one of the major global health challenges of the 21st century. Sedentary habits, unhealthy dietary patterns, and adverse environmental exposures have collectively contributed to the alarming worldwide increase in obesity and diabetes prevalence. However, the transition from normoglycemia to overt diabetes is highly heterogeneous, both clinically and biologically, involving metabolic, genetic, and environmental factors [1]. This progression typically passes through an intermediate state known as prediabetes, a particular state that encompasses a broad spectrum of metabolic and inflammatory states rather than a uniform intermediate condition [2,3]. Moreover, this heterogeneity extends beyond the distinct dysglycemic stages, showing differences also within each category itself. For instance, individuals with similar degrees of obesity and comparable glycemic status may differ substantially in inflammatory burden, comorbidity profiles, and long-term metabolic risk [2,4,5]. Furthermore, individuals with severe obesity represent a unique population in which metabolic dysfunction, inflammation, and comorbidities frequently coexist. This setting allows detailed phenotyping across a wide range of glycemic states while controlling for extreme adiposity. In addition, metabolic liver disease, including steatohepatitis, represents an additional inflammatory axis that may confound or interact with glycemia-related signals and therefore requires careful consideration [6,7,8].

Chronic low-grade inflammation is a hallmark of obesity and plays a central role in the pathogenesis of insulin resistance and type 2 diabetes mellitus (T2DM) [9,10,11]. Adipose tissue excess promotes immune cell infiltration, becoming a source of release for pro-inflammatory mediators contributing to systemic inflammatory activation, which in turn affects glucose homeostasis, vascular function, and causes organ-specific complications [12,13,14]. Circulating inflammatory mediators have therefore been extensively studied as potential biomarkers and mechanistic links between obesity and glycemic dysregulation. Nevertheless, most studies have focused on a limited number of classical cytokines, such as C-reactive protein or interleukin-6, and have often relied on categorical comparisons between diabetes stages [15,16].

Recent advances in affinity-based proteomics now enable the simultaneous quantification of dozens of inflammatory proteins in biological samples, providing a more comprehensive view of immune and inflammatory signaling in metabolic disease [17]. Such approaches have revealed that inflammatory alterations in T2DM extend beyond canonical cytokines and involve coordinated changes through chemokines, growth factors, and immune-regulatory pathways [16,18]. Importantly, these multidimensional inflammatory profiles often show substantial inter-individual variability, suggesting that distinct inflammatory phenotypes may coexist within conventional clinical categories [19]. Analytical strategies capable of capturing this complexity are therefore needed. While differential expression analyses and linear dimensionality reduction methods, such as principal component analysis (PCA), are informative, they may fail to detect nonlinear structures and subgroup-level patterns in high-dimensional proteomic data. Topological data analysis (TDA) is an unsupervised, network-based approach that preserves local and global relationships within complex datasets and has been successfully applied to identify disease subtypes and molecular phenotypes in heterogeneous clinical populations [20,21,22]. In metabolic disease, TDA offers a framework to uncover inflammatory states that transcend traditional diagnostic labels and may better reflect underlying biological diversity.

In the present exploratory study, we aimed to characterize circulating inflammatory protein profiles associated with glycemic status in individuals with severe obesity. Using proximity extension assay-based (PEA) proteomics, we first identified inflammatory proteins differentially expressed across normoglycemia, prediabetes, and T2DM and examined their distributions at the individual level. We then applied unsupervised topological data analysis to determine whether multidimensional inflammatory protein patterns define distinct patient subgroups beyond conventional glycemic classification. By combining proteomic data, clinical variables, and network analysis, this study provides a hypothesis-generating view of inflammatory heterogeneity in obesity-related dysglycemia.

## 2. Results

### 2.1. Clinical and Biochemical Characteristics of the Study Cohort

A total of 142 individuals were included in the proteomics sub-cohort and stratified according to glycemic status into normoglycemia (noDM, *n* = 69), prediabetes (PreDM, *n* = 25), and type 2 diabetes mellitus (T2DM, *n* = 48). As shown in Table 1, age differed significantly throughout groups (overall *p* < 0.05), with both PreDM and T2DM participants being older than noDM individuals, while no significant age difference was observed between PreDM and T2DM.

Sex distribution varied across glycemic categories, with a lower proportion of females in the T2DM group than in noDM (*p* < 0.001). BMI and waist circumference did not differ significantly among groups. As expected by the study design, FPG increased progressively across categories (noDM vs. PreDM and noDM vs. T2DM, both *p* < 0.001), whereas the difference between PreDM and T2DM did not reach statistical significance. HbA1c levels were significantly higher in T2DM than in both noDM and PreDM (both *p* < 0.001). Lipid parameters, liver enzymes, and C-reactive protein did not differ significantly across glycemic groups. MASLD severity and fibrosis stage were comparable across groups by selection strategy.

### 2.2. Differential Expression of Inflammatory Proteins Through Glycemic Status

**Figure 1:**
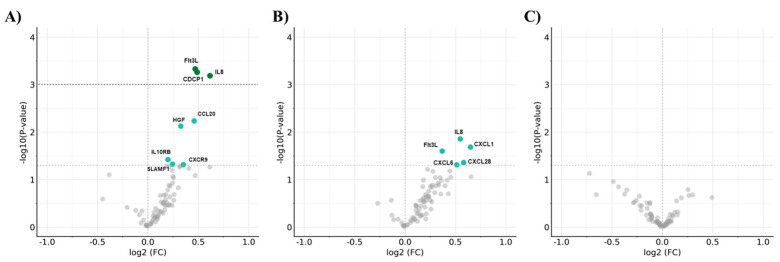
Differential expression of circulating inflammatory proteins according to glycemic status. Volcano plots illustrate the differential expression of plasma inflammatory proteins measured via proximity extension assay among glycemic stages. In (**A**) T2DM vs. noDM, (**B**) PreDM vs. noDM, and (**C**) T2DM vs. PreDM, Differential protein expressions using log2 fold change (log2 Fold Change) in NPX values between glycemic groups were plotted against negative log10 *p* values. The dashed horizontal line indicates the threshold for statistical significance after Benjamini–Hochberg false discovery rate (FDR) correction, which was used as the primary criterion for significance. Each point corresponds to one protein. Dark green points indicate proteins that remain significant after FDR-correction, while light green points represent proteins reaching nominal significance (unadjusted *p* < 0.05) but not when adjusted through multiple testing correction. Selected proteins with the largest effect sizes and/or strongest statistical support are labeled. Detailed statistics for all proteins are reported in Table S2.

Plasma inflammatory protein profiling identified multiple proteins differentially expressed across glycemic categories (Figure 1; Table S2). In the comparison between T2DM and noDM, Interleukin-8 (*CXCL8* gene) showed the strongest increase in plasma levels (log2FC = 0.62, FDR-adjusted *p* < 0.01). Fms-related tyrosine kinase 3 ligand (*FLT3LG* gene) and CUB domain-containing protein 1 (*CDCP1* gene) were also significantly upregulated in T2DM (log2FC = 0.47 and 0.49, respectively; both FDR-adjusted *p* < 0.01), representing the most robust findings after correction for multiple testing.

Additional proteins shown a trend of increases in T2DM compared with noDM, including C-C motif chemokine 20 (*CCL20* gene; log2FC = 0.46, *p* < 0.05), C-X-C motif chemokine 9 (*CXCL9* gene; log2FC = 0.35, *p* < 0.05), hepatocyte growth factor (*HGF* gene; log2FC = 0.33, *p* < 0.05), interleukin-10 receptor subunit beta (*IL10RB* gene; log2FC = 0.20, *p* < 0.05), and signaling lymphocytic activation molecule (*SLAMF1* gene; log2FC = 0.25, *p* < 0.05); however, these changes did not remain significant after FDR correction.

In contrast, the comparison between PreDM and noDM revealed fewer differences. Several proteins, including those encoded by IL-8, Flt3L, CXCL1, CXCL6, and CCL28 genes, showed nominal increases (unadjusted *p* < 0.05), but none remained significant after correction for multiple testing. No proteins were significantly different in the comparison between PreDM and T2DM based on FDR-adjusted and unadjusted *p*-values.

### 2.3. Individual-Level Distribution of Key Inflammatory Proteins

**Figure 2:**
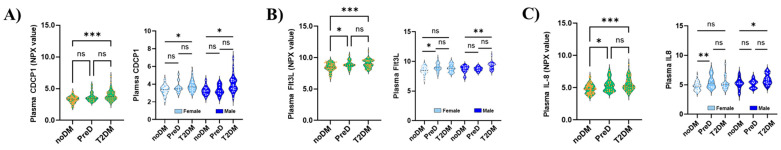
NPX distributions of CDCP1, Flt3L, and IL-8 among glycemic categories and stratified according to sex. Dot plots showing individual plasma NPX values for the most significantly differentially expressed proteins in plasma samples according to glycemic groups (noDM, PreDM, T2DM). In (**A**) CDCP1 NPX values, (**B**) Flt3L NPX values and (**C**) IL-8 NPX values. Each dot represents the NPX value for the protein in the plasma of each individual. Inside violin plots, horizontal dashed lines indicate median values with interquartile ranges. Panels are stratified by sex to illustrate sex-related variability. Significance was achieved at a *p*-value less than 0.05. *** *p* < 0.001; ** *p* < 0.01 and * *p* < 0.05; *ns*, not significant.

The distributions of IL-8, Flt3L, and CDCP1 NPX values across glycemic categories are shown in Figure 2. Median NPX values for all three proteins increased stepwise from noDM to PreDM and were highest in T2DM, as visualized in the volcano plots showing differential expression data. Despite these group-level differences, substantial overlap was observed across glycemic categories, with wide inter-individual variability in all groups. Sex-stratified visualization showed that these trends were present in both women and men, although variability was greater among men, particularly within the T2DM group.

### 2.4. Topological Data Analysis Identifies Inflammatory Protein-Defined Clusters

**Figure 3:**
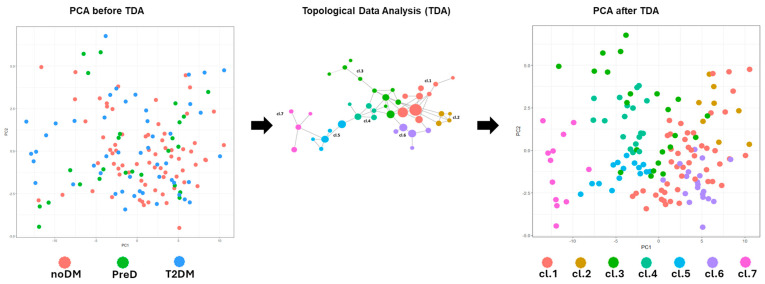
Topological data analysis (TDA) of inflammatory proteomic profiles. TDA network constructed from plasma inflammatory protein expression profiles. Each node represents one or more individuals with similar multidimensional protein expression patterns, and edges represent similar relationships. In principal component analysis (PCA) after TDA, nodes are colored according to the seven distinct clusters (cl1–cl7) identified, reflecting heterogeneous inflammatory phenotypes. (PCA) projection is shown for comparison, highlighting the limited separation of individuals by glycemic status using linear dimensionality reduction.

Principal component analysis did not clearly separate individuals according to glycemic status (Figure 3). In contrast, TDA organized samples into a structured network of seven clusters (cl1–cl7) based solely on inflammatory protein expression patterns. From a methodological perspective, TDA has been used in biomedicine to represent complex patient similarity structures as networks that can reveal subgroups and gradients that are not readily visible with linear projections. In type 2 diabetes specifically, topology-based patient similarity approaches have identified distinct subgroups with differing clinical and biological characteristics, supporting the use of topology as an exploratory stratification tool. We therefore used TDA to organize inflammatory proteomic heterogeneity rather than to define definitive disease subtypes.

Cluster sizes and glycemic composition are summarized in Table 2. Cluster cl7 showed the highest proportion of T2DM individuals (58.3% T2DM, 33.3% PreDM, 8.3% noDM), whereas clusters cl1 and cl2 were predominantly composed of normoglycemic individuals. PreDM participants were distributed among multiple clusters and did not form a distinct inflammatory group.

### 2.5. Cluster-Specific Inflammatory Protein Patterns

**Figure 4:**
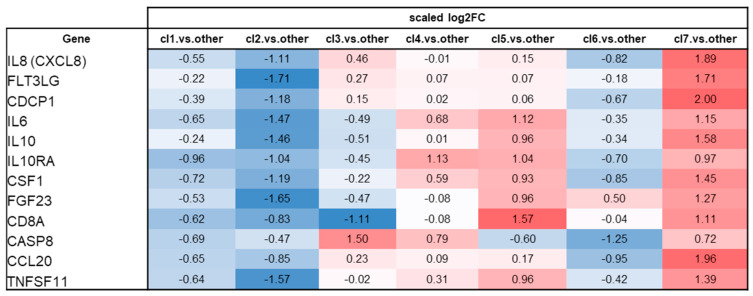
Cluster-specific inflammatory protein signatures identified through topological data analysis. Cluster-specific inflammatory protein signatures across TDA-defined clusters (cl1–cl7). Heatmap showing scaled log2 fold-change values for selected inflammatory proteins in the TDA-defined clusters (cl1–cl7). Values represent relative protein expression in each cluster compared with the remainder of the cohort. Red indicates higher relative expression, and blue indicates lower relative expression.

Figure 4 shows the expression patterns throughout the TDA-defined clusters for selected inflammatory proteins. The full protein signatures for each cluster are reported numerically in Table S3. Cluster cl7 was characterized by marked upregulation of several inflammatory proteins relative to the cohort mean, including IL-8 (scaled log2FC = +1.89), CDCP1 (+2.00), and Flt3L (+1.71). Additional proteins upregulated in cl7 included IL-10 (+1.58), CSF-1 (+1.45), FGF23 (+1.27), IL-6 (+1.15), CD8A (+1.11), and IL10RA (+0.97), indicating coordinated activation of chemokine, cytokine, immune cell-associated, and regulatory pathways.

Other clusters exhibited distinct inflammatory profiles. Clusters cl1 and cl2 showed generally lower expression of inflammatory proteins, with negative or near-zero scaled log2FC values across most markers. Clusters cl3 and cl4 displayed selective enrichment of proteins related to apoptotic and stress signaling, including CASP8, without concurrent upregulation of the IL-6/IL-10 axis. Clusters cl5 and cl6 exhibited intermediate inflammatory profiles.

### 2.6. Clinical Characteristics Across TDA-Defined Clusters

**Figure 5:**
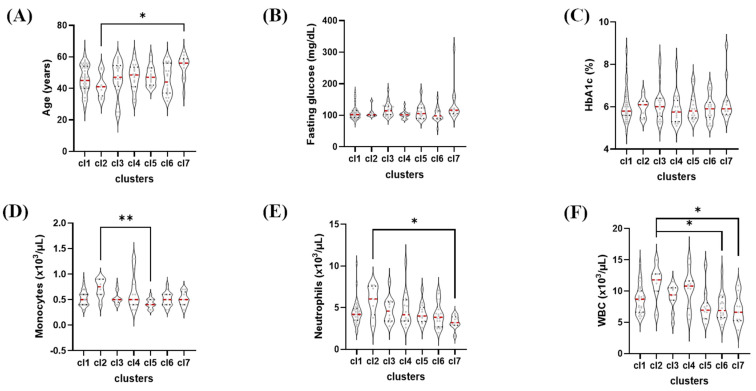
Distribution of glycemic and metabolic variables across TDA-defined clusters (cl1–cl7). Violin plots in the panels show (**A**) age, (**B**) fasting plasma glucose (FPG), (**C**) glycated hemoglobin (HbA1c), (**D**) monocyte counts, (**E**) neutrophil counts, and (**F**) total white blood cell counts among clusters. Data are presented as individual values with median and interquartile ranges. Median values are indicated by red horizontal lines within each violin plot. Significance was achieved at a *p*-value less than 0.05. ** *p* < 0.01 and * *p* < 0.05.

Clinical and laboratory characteristics through clusters are summarized in Table 2 and visualized in Figure 5. Overall heterogeneity across clusters was observed for several variables, including age, fasting plasma glucose, and HbA1c; however, these differences did not remain significant after post hoc correction for multiple comparisons.

In contrast, white blood cell count differed significantly across clusters after multiple testing correction. Clusters cl6 and cl7 exhibited significantly lower white blood cell counts compared with clusters characterized by lower inflammatory protein expression. A similar pattern was observed for neutrophil counts. BMI and MASLD severity did not differ significantly across clusters, consistent with the study design. The prevalence of at least one comorbidity differed significantly across clusters, with higher comorbidity burden observed in inflammatory-enriched clusters, including cl7.

### 2.7. Multivariable and Sensitivity Analyses of Key Inflammatory Proteins

Multivariable linear regression analyses were performed for six pre-specified proteins (IL-8, Flt3L, CDCP1, IL-6, IL-10, and CSF-1), adjusting for age, sex, BMI, MASLD stage, antidiabetic therapy, and medication burden score (Tables S4–S9).

When glycemic status was modeled categorically, none of the proteins showed an independent association with glycemic category after adjustment. IL-8 levels were independently associated with sex (β = −0.53 NPX, *p* = 0.0085) and medication burden score at the model level (*p* = 0.041). Flt3L was strongly associated with age (β = 0.033 NPX per year, *p* < 0.0001) and sex (β = −0.30 NPX, *p* = 0.030). CDCP1 showed no independent association with categorical glycemic status, with a borderline association with medication burden score (*p* = 0.067). Among secondary markers, IL-6 was independently associated with age (β = 0.027 NPX per year, *p* = 0.0055) and BMI (β = 0.051 NPX per kg/m^2^, *p* = 0.0020). IL-10 was not associated with glycemic status but was associated with medication burden score (*p* = 0.016). CSF-1 was independently associated with age (β = 0.011 NPX per year, *p* = 0.015), MASLD stage (no MASLD vs. MASLD, β = 0.25 NPX, *p* = 0.025), and medication burden score (*p* = 0.006).

In sensitivity analyses modeling fasting plasma glucose as a continuous variable, FPG was independently associated with IL-8 (β = 0.008 NPX per mg/dL, *p* = 0.005) and CDCP1 (β = 0.010 NPX per mg/dL, *p* < 0.0001), but not with Flt3L (*p* = 0.27). These coefficients indicate that, across a clinically relevant 50 mg/dL difference in FPG, IL-8 and CDCP1 levels increase by approximately 0.4–0.5 NPX. This supports a modest but biologically meaningful association with glycemic burden, rather than a strong correlation suitable for immediate biomarker application.

## 3. Discussion

In this pilot proteomic study involving severely obese subjects, we combined differential expression analysis with unsupervised topological data analysis to characterize inflammatory heterogeneity across glycemic stages. Three main conclusions emerge. First, specific inflammatory proteins, most prominently IL-8 (CXCL8), Flt3L (FLT3LG), and CDCP1, were the most consistently associated proteins in unadjusted group-level analyses comparing T2DM with normoglycemia, although categorical associations were attenuated after multivariable adjustment. Second, unsupervised TDA revealed inflammatory proteomic phenotypes that were only partially aligned with conventional glycemic classification, highlighting substantial biological heterogeneity. Third, inflammatory protein-defined clusters differed in clinical burden and systemic inflammatory markers despite minimal differences in glycemic measures after correction for multiple comparisons. Together, these findings support preliminary evidence of inflammatory proteomic heterogeneity in severe obesity, indicating that glycemia-associated protein changes occur within a broader landscape of inter-individual inflammatory variation and providing a hypothesis-generating basis for future validation in larger, longitudinal cohorts. Differential expression analysis identified IL-8 as the most strongly increased inflammatory protein in T2DM relative to normoglycemia. These effect sizes are modest, consistent with chronic low-grade inflammation rather than acute inflammatory responses, and remained significant after FDR correction in unadjusted differential expression analyses. In contrast, no inflammatory proteins reached adjusted significance in comparisons involving prediabetes. This likely reflects both smaller effect sizes at this stage of dysglycemia and the biological heterogeneity encompassed within the prediabetic category.

Importantly, the TDA-derived clusters should be interpreted as exploratory molecular strata rather than clinically validated “phenotypes”. In our cohort, standard clinical measures, including fasting glucose and HbA1c, did not robustly differentiate clusters after correction, indicating limited clinical anchoring. This dissociation is biologically plausible: inflammatory activation in severe obesity can reflect multiple overlapping axes (adipose dysfunction, immune trafficking, liver disease biology, aging, comorbidity burden, etc.) that are not captured by categorical glycemic staging. In line with the concept of metabolically healthy versus unhealthy obesity and related proteomic phenotypes, individuals may exhibit markedly different inflammatory burdens despite similar glycemic status [23,24].

Visualization of individual NPX distributions revealed substantial overlap across glycemic groups for all three proteins, despite higher median values in T2DM. Notably, some normoglycemic and prediabetic individuals exhibited inflammatory protein levels comparable to those observed in T2DM. This overlap indicates that elevated inflammatory signaling is not restricted to individuals with overt diabetes and supports the need for analytical approaches that do not rely exclusively on categorical glycemic definitions. In this context, TDA provided added value by organizing individuals into clusters based on multidimensional inflammatory protein expression rather than predefined clinical labels [19]. TDA did not identify additional candidate proteins beyond those highlighted by differential expression analysis; instead, it contextualized IL-8, Flt3L, and CDCP1 within coordinated inflammatory phenotypes. The T2DM-enriched cluster (cl7) showed marked upregulation of these three proteins at the cluster level, together with increased expression of additional cytokine and immune-related proteins, including IL-10, CSF-1, IL-6, CD8A, and IL10RA. This pattern reflects coordinated activation of chemokine, cytokine, immune cell-associated, and regulatory pathways rather than isolated biomarker changes.

Prediabetic individuals were distributed across multiple clusters and did not form a discrete inflammatory group, reinforcing the concept that prediabetes represents a biologically heterogeneous state rather than a uniform intermediate stage between normoglycemia and T2DM [25]. This observation aligns with the lack of robust differential expression involving the prediabetic group and cautions against interpreting cross-sectional comparisons as reflecting linear disease progression.

Despite pronounced differences in inflammatory protein expression across clusters, glycemic measures such as fasting plasma glucose and HbA1c did not differ significantly between clusters after post hoc correction, indicating that the inflammatory phenotypes captured by TDA are not simply proxies for glycemic severity. Notably, clusters characterized by higher expression of inflammatory proteins, including IL-8, Flt3L, and CDCP1, did not exhibit corresponding increases in circulating leukocyte counts and, in some cases, showed lower neutrophil levels. Although this may appear counterintuitive, circulating leukocyte counts do not necessarily mirror the degree of inflammatory signaling in chronic metabolic disease, where low-grade inflammation is primarily driven by tissue-level immune activation rather than by expansion of circulating immune cells [26] In obesity, T2DM, and MASLD, metabolically active tissues such as adipose tissue and liver act as major sources of cytokines and chemokines, contributing to systemic inflammatory signaling independently of peripheral leukocyte counts [27,28]. In addition, chemokine-driven pathways, including IL-8-mediated signaling, promote recruitment and retention of immune cells within inflamed tissues, resulting in a relative dissociation between circulating cell numbers and tissue-level immune activity [29,30]. Consistent with this framework, inflammatory-enriched clusters in our cohort also showed a higher prevalence of comorbidities, suggesting that the observed proteomic signatures capture broader systemic disease complexity rather than glycemic status alone. Because circulating leukocyte counts are further influenced by comorbidity burden, medication exposure, and age-related immune remodeling, these hematologic differences should be interpreted as markers of systemic heterogeneity rather than evidence for a single mechanistic pathway [31,32].

Multivariable regression analyses further refined the interpretation of these findings. After adjustment for age, sex, BMI, MASLD stage, antidiabetic therapy, and medication burden score, categorical glycemic status was no longer independently associated with IL-8, Flt3L, or CDCP1. This attenuation indicates that crude group-level differences are influenced by demographic and clinical context and should not be interpreted as evidence that these proteins are independent markers of dysglycemia. However, sensitivity analyses modeling fasting plasma glucose as a continuous variable revealed independent associations with IL-8 and CDCP1, but not with Flt3L. These results suggest that certain inflammatory signals scale with glycemic burden rather than diagnostic thresholds, whereas others, such as Flt3L, appear more strongly linked to age- and sex-related immune processes. The attenuation of associations when glycemic status is modeled categorically underscores that inflammatory signals in severe obesity are shaped by broader context. Age-related immune remodeling (inflammaging) is a major determinant of circulating immune mediators and proteomic profiles, and sex-associated immune differences are also well documented [33]. These established influences provide a biological basis for why age and sex can explain variance in inflammatory proteins that is not captured by glycemic categories alone [34,35].

Recent literature provides strong external support for the association of IL-8, Flt3L, and CDCP1 with diabetes and dysglycemia, reinforcing the biological plausibility of our findings. Circulating IL-8 has been consistently reported to be elevated in individuals with T2DM and to correlate with adverse inflammatory and cardiometabolic profiles, including higher fasting glucose, HbA1c, LDL-cholesterol, and pro-inflammatory cytokines, alongside lower adiponectin and vitamin D levels [36,37,38,39]. These associations support IL-8 as a marker of coordinated immune-metabolic dysfunction rather than isolated hyperglycemia. Large Olink-based proteomics studies have similarly identified CDCP1 as a key inflammatory protein associated with glycemic control, insulin resistance, and diabetes-related complications across both type 1 and type 2 diabetes, with CDCP1 clustering alongside stress- and inflammation-related proteins such as FGF21 and HGF. Beyond diabetes, multicentric validation studies have demonstrated strong associations between soluble CDCP1 and obesity-related steatohepatitis, visceral adiposity, dyslipidemia, and metabolic syndrome, and mechanistic work has linked CDCP1 signaling to pathways involved in hepatic inflammation and fibrosis [38]. Flt3L has also emerged from targeted proteomics studies as an inflammatory protein associated with cardiovascular disease development in T2DM, suggesting a role in immune activation and hematopoietic stress that extends beyond glycemic regulation alone. Collectively, these findings indicate that IL-8 [37], Flt3L [39], and CDCP1 [38] capture overlapping but distinct dimensions of immune-metabolic dysfunction in diabetes and support their interpretation as components of coordinated inflammatory phenotypes rather than isolated disease markers.

Several limitations should be acknowledged for this exploratory study. The cross-sectional design precludes inference regarding temporal sequence, causality, or progression from prediabetes to T2DM, and the relatively small size of the prediabetic group limits statistical power. The proteomics sub-cohort was intentionally selected and not randomly sampled, which may limit generalizability. Proteomic measurements were relative rather than absolute. An additional limitation concerns pre-analytical conditions. Plasma samples used for proteomic profiling were collected in a non-fasting state, whereas routine biochemical measurements were obtained from fasting blood samples. This may have increased biological variability in circulating protein levels and complicates direct comparison between proteomic and standard laboratory parameters. Nevertheless, all proteomic samples were collected, processed, and stored according to the same standardized procedure across glycemic groups, thereby reducing the likelihood of systematic between-group bias. Even so, pre-analytical effects on protein measurements cannot be completely excluded. The study did not include longitudinal outcomes or external validation; therefore, the observed proteomic patterns should be regarded as exploratory and hypothesis-generating, without immediate predictive or clinical utility.

In conclusion, inflammatory proteomic profiling in severe obesity reveals substantial heterogeneity that is not fully captured by conventional categorical glycemic classification. IL-8, Flt3L, and CDCP1 emerge as key components in the inflammatory signatures and the most consistently associated proteins in unadjusted group-level analyses, but their association with categorical glycemic status were attenuated after multivariable adjustment. Thus, these proteins should not be interpreted as robust independent markers of dysglycemia based on the current cross-sectional analysis. The inflammatory proteomic phenotypes identified herein provide a hypothesis-generating framework for future longitudinal and interventional studies. Whether such system-level patterns can support risk stratification, prediction of metabolic deterioration, or clinically meaningful subgrouping remains hypothetical and requires external validation in larger prospective cohorts.

## 4. Materials and Methods

### 4.1. Patient Enrollment and Diagnosis

This study is a cross-sectional analysis of a prospectively enrolled bariatric cohort. A total of 351 individuals with obesity were enrolled in the bariatric surgery program at the Surgical Department of Cattinara Hospital. Inclusion and exclusion criteria have been described previously [40]. All participants provided written informed consent, and all sensitive data were anonymized. The study protocol was approved by the local Ethics Committee (Protocol No. 22979) and registered with ClinicalTrials.gov (NCT06098417).

Glycemic status was defined according to WHO criteria using fasting plasma glucose (FPG) [41]. Prediabetes was defined as FPG ≥ 110 and <126 mg/dL. Diabetes mellitus was defined as FPG ≥ 126 mg/dL, current antidiabetic therapy, or a diagnosis of diabetes within the previous 12 months. In three individuals, FPG was unavailable, and glycemic classification was based on HbA1c according to ADA criteria [42]. Liver biopsies were obtained intraoperatively during laparoscopic surgery. Hepatic steatosis was graded on hematoxylin and eosin-stained sections. Samples with <5% steatosis and no evidence of hepatocellular injury or fibrosis were considered histologically normal, whereas samples with ≥5% steatosis were classified as MASLD. Also, steatohepatitis and fibrosis were defined histologically according to the Kleiner–Brunt criteria, grading steatosis, lobular/portal inflammation and ballooning, and staging fibrosis in each biospecimen [43].

Selection of the proteomics sub-cohort was guided by stratification according to glycemic status. Because the prediabetic group (PreDM) represented the smallest subgroup in the overall cohort (*n* = 28), all eligible individuals were initially considered; however, plasma samples from three participants were excluded due to haemolysis prior to proteomic analysis and sub-cohort construction, resulting in 25 PreDM individuals. Normoglycemic (noDM) and type 2 diabetes mellitus (T2DM) participants were subsequently selected to achieve comparable distributions of sex and MASLD severity, including the presence or absence of fibrosis, across glycemic groups.

This selection strategy resulted in a proteomics sub-cohort of 143 individuals. During proteomic quality control, one sample failed the internal Olink quality criteria, and the corresponding individual was excluded. The final analytical cohort, therefore, comprised 142 individuals, including 69 noDM, 25 PreDM, and 48 T2DM, who were used in all downstream analyses. Detailed distributions of glycemic status, sex, and MASLD categories in the full cohort are reported in Table S1, and a flow diagram showing selection of the proteomic sub-cohort from the full bariatric surgery cohort is presented as Figure S1.

### 4.2. Routine Blood Measurements and Plasma Proteomics

Anthropometric measurements (age, sex, body weight, height, BMI, and waist circumference) were recorded at a baseline visit approximately two weeks before surgery. Fasting blood samples were collected by venipuncture into K2EDTA tubes (Vacuette^®^, Greiner Bio-One, Kremsmünster, Austria) for routine laboratory analyses. Standard biochemical parameters, including glucose, alanine aminotransferase (ALT), aspartate aminotransferase (AST), gamma-glutamyl transferase (GGT), total bilirubin, albumin, platelet count, and lipid profile, were measured using a Cobas 6000 analyzer (Roche Diagnostics, Indianapolis, IN, USA).

For proteomic analyses, non-fasting blood samples were collected using K2EDTA tubes, maintained at 4 °C for 16–18 h, and then plasma was separated. Samples were centrifuged at 2000× *g* for 10 min at 4 °C, followed by a second centrifugation of the supernatant at 6000× *g* for 5 min. Plasma was aliquoted and stored at −80 °C until analysis.

### 4.3. Proteome Profiling and Data Processing

Plasma inflammatory proteins were quantified using the Olink^®^ Inflammation panels on the Olink Signature Q100 platform (Olink Proteomics AB, Uppsala, Sweden), based on PEA technology. In brief, target proteins are recognized by pairs of oligonucleotide-labeled antibodies, enabling subsequent DNA amplification and quantification by means of microfluidic real-time PCR [44]. Raw cycle threshold (Ct) values underwent quality control and normalization using internal and external controls with Olink NPX Signature Software (v1.13.0). Protein abundance is reported as Normalized Protein Expression (NPX), an arbitrary unit on a log2 scale, with higher NPX values indicating higher protein levels.

Data exploration and initial analyses were performed using the Olink web-based analysis platform (https://olink.com/software/olink-analyze, accessed on 2 October 2025). A total of 178 samples from 143 individuals were analyzed across two Olink plates. One sample failed quality control and was excluded. Batch effects were corrected using a two-step strategy in Rstudio v.2024.12.0+467, through standard bridge normalization [45] and the Bamboo algorithm [46], leveraging 35 technical replicate samples (12 noDM, 12 PreDM, and 11 T2DM) processed on both plates as shared bridging controls. Consistent with previous antibody-based proteomics studies [47], proteins with more than 20% missing values were excluded, resulting in a final dataset of 74 inflammatory proteins (out of 92 total proteins in the Olink panel) included in downstream analyses. *p*-values derived from *t*-tests were adjusted for multiple testing using the Benjamini–Hochberg method, with adjusted *p*-values < 0.05 considered statistically significant. See Supplementary Methods for more details (Figure S2 and batch effect correction section).

### 4.4. Topological Data Analysis

Building topological models as networks, TDA allows the identification of specific connected clusters of patients sharing similar features (called ‘communities’) in a graph-based data representation. Proteomics data were used to build the TDA graph. Regarding TDA parameters, the first two dimensions extracted from the PCA were chosen as lens functions to guide network construction. These components were used solely for data organization and not for clustering, which was performed on the full proteomic dataset.

TDA parameters, including the number of bins (NB) and the bin overlapping ratio (BO), were selected via a ‘grid search’ approach to obtain a stable and interpretable scale-free network structure. The best parameter set was NB = 5 and OB = 20%. Topological data analysis was performed using the ‘PIUMA’ Bioconductor package [48]. Networks have been built using Cytoscape 3.10.4 [49]; each node represents a group of samples with similar multivariate proteomic profiles, while edges indicate shared samples between nodes (quantified using the Jaccard Index). The Girvan–Newman algorithm implemented in the ‘clustermaker2’ Cytoscape plug-in was used to identify communities in the network [50]. Communities: The community’s robustness was assessed via bootstrap resampling (100 iterations) using the clusterboot function (fpc R package 2.2-14).

### 4.5. Statistical Analysis

Participants were stratified according to diabetes stage. Statistical comparisons among noDM, PreDM, and T2DM groups were performed for both clinical variables and NPX protein data. Continuous variables are presented as mean ± standard deviation (SD) or median with interquartile range (IQR), as appropriate, while categorical variables are reported as counts and percentages. Normality of continuous variables was assessed using the D’Agostino—Pearson omnibus test. Group differences for normally distributed variables were analyzed using independent *t*-tests or one-way analysis of variance (ANOVA), whereas non-normally distributed variables were analyzed using Mann–Whitney U or Kruskal–Wallis tests with appropriate post hoc analyses. Categorical variables were compared using chi-square tests with correction when required.

Multivariable linear regression analyses were performed to evaluate independent associations between selected inflammatory proteins and glycemic status while accounting for potential confounders. Glycemic status was included as a categorical independent variable (noDM, PreDM, and T2DM), with normoglycemia used as the reference category. All models were adjusted for age, sex, BMI, MASLD stage, antidiabetic therapy (yes/no), and medication burden score (calculated as the number of concomitant medications into the categories: 0, 1, and ≥2 medications). To assess whether inflammatory protein levels were associated with the degree of glycemic dysregulation rather than diagnostic category, sensitivity analyses were conducted replacing glycemic status with FPG as a continuous independent variable, using otherwise identical covariate adjustment. Model assumptions were evaluated through assessment of residual normality (D’Agostino—Pearson omnibus, Shapiro—Wilk, and Kolmogorov–Smirnov tests) and multicollinearity using variance inflation factors (VIF). Regression coefficients (β), 95% confidence intervals, and two-sided *p*-values are reported, with *p* < 0.05 considered statistically significant. All statistical analyses were performed using GraphPad Prism (version 10.6.1; GraphPad Software, San Diego, CA, USA).

## Figures and Tables

**Table 1:** Clinical and biochemical characteristics of the study cohort by glycemic status.

	Morbidly Obese Cohort (*n* = 142)			*p-*Value		
Clinical Variables	NoDM (*n* = 69)	PreDM (*n* = 25)	T2DM (*n* = 48)	noDM vs. PreDM	noDM vs. T2DM	PreDM vs. T2DM
Age (years)	44.1 ± 8.2	53.0 ± 9.4	58.1 ± 9.3	0.002	0.022	0.72
Gender female n, (%)	61, (88%)	17, (68%)	26, (54%)	0.02	0.0001	0.25
BMI (kg/m^2^)	43.2 ± 4.8	44.6 ± 6.4	43.9 ± 5.3	0.33	0.98	0.99
Fasting glucose (mg/dL)	98.9 ± 14.8	115.2 ± 7.0	125.1 ± 38.0	0.0001	0.0001	0.99
AST (U/L)	23.4 ± 10.6	25.0 ± 8.2	28.0 ± 16.8	0.54	0.48	0.99
ALT (U/L)	29.5 ± 22.5	29.8 ± 11.4	34.5 ± 24.5	0.53	0.23	0.99
GGT (U/L)	38.4 ± 53.9	47.8 ± 42.1	39.8 ± 25.6	0.02	0.02	0.99
Total bilirubin (mg/dL)	0.62 ± 0.29	0.70 ± 0.30	0.61 ± 0.25	0.72	0.99	0.76
Alkaline phosphatase (U/L)	83.7 ± 27.4	92.1 ± 28.1	89.7 ± 23.5	0.25	0.18	0.99
Triglycerides (mg/dL)	123.0 ± 51.1	160.0 ± 83.7	162.8 ± 72.1	0.16	0.003	0.99
Tot. Cholesterol (mg/dL)	209.4 ± 34.6	223.0 ± 38.5	208.3 ± 44.5	0.41	0.99	0.33
HDL Cholesterol (mg/dL)	50.3 ± 9.9	47.6 ± 10.8	45.8 ± 10.1	0.99	0.03	0.90
Ferritin (ng/mL)	56.3 ± 55.3	68.2 ± 54.3	93.1 ± 78.7	0.31	0.011	0.99
Transferrin (mg/dL)	287.0 ± 55.3	274.4 ± 40.2	286.4 ± 37.8	0.66	0.99	0.6
Glycated haemoglobin (%)	5.7 ± 0.5	5.98 ± 0.4	6.50 ± 0.9	0.05	<0.0001	0.19
Platelets (×10^−3^/µL)	254 ± 61	239.4 ± 48	264.6 ± 64	0.850	0.62	0.14
Lymphocytes (×10^9^/L)	2.2 ± 0.6	1.9 ± 0.5	2.1 ± 0.6	0.29	0.99	0.6
Neutrophils (×10^9^/L)	4.4 ± 1.5	4.4 ± 1.4	6.3 ± 9.5	0.99	0.99	0.99
Monocytes (×10^9^/L)	0.6 ± 0.2	0.5 ± 0.2	0.5 ± 0.2	0.99	0.99	0.99
White blood cells (×10^9^/L)	8.7 ± 2.8	8.8 ± 2.2	8.3 ± 2.6	0.99	0.99	0.99
Liver fibrosis 0/1 (%)	51%/44%	32%/68%	46%/54%	0.11	0.60	0.25
No MASLD/MASLD/MASH, (%)	27%/43%/30%	16%/40%/44%	12%/46%/42%	0.39	0.16	0.86

Clinical and biochemical characteristics of the morbidly obese cohort stratified by glycemic status: normoglycemia/no diabetes (NoDM, *n* = 69), prediabetes (PreDM, *n* = 25), and type 2 diabetes mellitus (T2DM, *n* = 48). Continuous variables are presented as mean ± standard deviation and categorical variables as n (%). *p*-values correspond to pairwise comparisons between groups (NoDM vs. PreDM, NoDM vs. T2DM, and PreDM vs. T2DM). Abbreviations: BMI, body mass index; AST, aspartate aminotransferase; ALT, alanine aminotransferase; GGT, gamma-glutamyl transferase; HDL, high-density lipoprotein; MASLD, metabolic dysfunction-associated steatotic liver disease; MASH, metabolic dysfunction-associated steatohepatitis.

**Table 2:** Clinical characteristics across TDA-defined inflammatory clusters.

	cl1	cl2	cl3	cl4	cl5	cl6	cl7	*p*-Value
	(*n* = 48)	(*n* = 8)	(*n* = 22)	(*n* = 18)	(*n* = 15)	(*n* = 19)	(*n* = 12)	
class								
noDM	27 (56.3%)	5 (62.5%)	6 (27.3%)	11 (61.1%)	8 (53.3%)	11 (57.9%)	1 (8.3%)	0.036
PreDM	3 (6.3%)	2 (25.0%)	7 (31.8%)	2 (11.1%)	4 (26.7%)	3 (15.8%)	4 (33.3%)	
T2DM	18 (37.5%)	1 (12.5%)	9 (40.9%)	5 (27.8%)	3 (20.0%)	5 (26.3%)	7 (58.3%)	
MASLD_stage								
MASH	18 (37.5%)	2 (25.0%)	12 (54.5%)	5 (27.8%)	2 (13.3%)	9 (47.4%)	4 (33.3%)	0.058
MASL	26 (54.2%)	5 (62.5%)	5 (22.7%)	6 (33.3%)	7 (46.7%)	7 (36.8%)	6 (50.0%)	
No MASL	4 (8.3%)	1 (12.5%)	5 (22.7%)	7 (38.9%)	6 (40.0%)	3 (15.8%)	2 (16.7%)	
class2								
noDM	27 (56.3%)	5 (62.5%)	6 (27.3%)	11 (61.1%)	8 (53.3%)	11 (57.9%)	1 (8.3%)	0.017
DM	21 (43.8%)	3 (37.5%)	16 (72.7%)	7 (38.9%)	7 (46.7%)	8 (42.1%)	11 (91.7%)	
class2_stage								
DM_MASH	10 (20.8%)	0 (0%)	11 (50.0%)	2 (11.1%)	1 (6.7%)	4 (21.1%)	3 (25.0%)	0.023
DM_MASL	10 (20.8%)	3 (37.5%)	3 (13.6%)	2 (11.1%)	4 (26.7%)	4 (21.1%)	6 (50.0%)	
DM_No_MASL	1 (2.1%)	0 (0%)	2 (9.1%)	3 (16.7%)	2 (13.3%)	0 (0%)	2 (16.7%)	
noDM_MASH	8 (16.7%)	2 (25.0%)	1 (4.5%)	3 (16.7%)	1 (6.7%)	5 (26.3%)	1 (8.3%)	
noDM_MASL	16 (33.3%)	2 (25.0%)	2 (9.1%)	4 (22.2%)	3 (20.0%)	3 (15.8%)	0 (0%)	
noDM_No_MASL	3 (6.3%)	1 (12.5%)	3 (13.6%)	4 (22.2%)	4 (26.7%)	3 (15.8%)	0 (0%)	
Age	45.0 [40.0, 54.0]	41.0 [37.8, 44.5]	47.0 [42.3, 53.8]	48.5 [41.8, 52.8]	47.0 [43.0, 52.0]	44.0 [37.5, 53.5]	56.0 [51.5, 58.3]	0.047
gender Male	11 (22.9%)	0 (0%)	10 (45.5%)	5 (27.8%)	4 (26.7%)	2 (10.5%)	6 (50.0%)	0.042
Commorbidities	24 (50.0%)	3 (37.5%)	18 (81.8%)	14 (77.8%)	10 (66.7%)	8 (42.1%)	11 (91.7%)	0.005
pharma_ther	19 (39.6%)	3 (37.5%)	16 (72.7%)	14 (77.8%)	9 (60.0%)	6 (31.6%)	11 (91.7%)	<0.001
anti_diabetic	15 (31.3%)	1 (12.5%)	7 (31.8%)	6 (33.3%)	3 (20.0%)	4 (21.1%)	7 (58.3%)	0.293
anti_dyslipd_hyperChol	6 (12.5%)	1 (12.5%)	6 (27.3%)	4 (22.2%)	2 (13.3%)	3 (15.8%)	3 (25.0%)	0.761
anty_Hypertens	11 (22.9%)	2 (25.0%)	10 (45.5%)	6 (33.3%)	3 (20.0%)	5 (26.3%)	7 (58.3%)	0.172
OSAS_ther	4 (8.3%)	1 (12.5%)	4 (18.2%)	5 (27.8%)	4 (26.7%)	1 (5.3%)	3 (25.0%)	0.245
Fasting_Glucose	102 [93.0, 114]	102 [99.0, 107]	114 [102, 129]	102 [97.3, 107]	105 [93.0, 123]	98.0 [90.5, 113]	116 [109, 131]	0.022
Glycated_haemoglobin	5.80 [5.60, 6.43]	6.10 [5.63, 6.15]	6.00 [5.63, 6.38]	5.75 [5.30, 6.10]	5.80 [5.53, 6.25]	5.90 [5.55, 6.15]	5.90 [5.75, 6.23]	0.864
Missing	0 (0%)	0 (0%)	0 (0%)	2 (11.1%)	1 (6.7%)	0 (0%)	0 (0%)	
Height	1.70 [1.60, 1.73]	1.70 [1.68, 1.73]	1.70 [1.60, 1.70]	1.60 [1.60, 1.78]	1.60 [1.60, 1.70]	1.70 [1.60, 1.70]	1.75 [1.70, 1.80]	0.410
Weight	120 [109, 146]	116 [112, 128]	126 [104, 138]	118 [104, 133]	127 [106, 137]	120 [113, 131]	128 [111, 137]	0.667
Ideal_weight	69.3 [65.2, 75.7]	68.5 [67.9, 75.2]	68.9 [64.0, 75.7]	63.2 [61.2, 75.5]	66.4 [64.0, 73.2]	69.7 [67.2, 71.4]	76.6 [70.4, 79.4]	0.295
BMI	43.0 [40.6, 48.0]	41.1 [38.0, 41.4]	44.5 [40.7, 46.9]	43.4 [42.4, 45.4]	43.9 [40.9, 47.8]	42.6 [41.0, 46.5]	43.1 [40.8, 46.6]	0.615
Excess_Weight	50.5 [42.3, 66.7]	44.4 [43.3, 45.4]	57.9 [40.0, 68.5]	50.9 [42.7, 58.7]	54.8 [42.8, 63.7]	50.4 [43.6, 60.3]	54.6 [41.1, 60.6]	0.673
AST	23.5 [19.0, 29.0]	23.0 [20.5, 24.3]	24.0 [18.3, 28.8]	20.5 [19.0, 25.5]	22.0 [19.5, 24.5]	21.0 [17.0, 25.5]	28.0 [20.5, 30.3]	0.636
ALT	26.5 [19.8, 34.3]	33.0 [20.0, 43.3]	26.5 [17.0, 34.5]	26.0 [19.5, 31.8]	24.0 [18.0, 26.5]	26.0 [16.5, 33.0]	30.0 [18.3, 51.0]	0.785
GGT	27.5 [21.0, 38.5]	28.0 [20.8, 40.0]	29.0 [21.3, 56.8]	32.0 [23.0, 43.8]	22.0 [15.0, 37.5]	24.0 [18.5, 26.0]	38.0 [22.0, 74.5]	0.302
Alkaline_Phosphatase	81.0 [69.8, 101]	87.5 [67.0, 102]	83.5 [69.5, 105]	77.0 [70.3, 111]	81.0 [71.0, 91.0]	83.0 [62.0, 96.5]	71.5 [62.8, 122]	0.989
Triglycerides	122 [105, 162]	130 [97.0, 210]	137 [100, 172]	115 [92.0, 162]	95.0 [79.0, 157]	130 [84.0, 149]	153 [137, 216]	0.374
Total_cholesterol	215 [183, 236]	198 [184, 217]	224 [155, 258]	213 [182, 237]	207 [172, 240]	204 [190, 228]	221 [205, 258]	0.780
Missing	0 (0%)	0 (0%)	0 (0%)	0 (0%)	1 (6.7%)	0 (0%)	0 (0%)	
HDL_cholesterol	45.0 [41.8, 51.0]	47.0 [41.5, 51.0]	48.0 [37.5, 54.8]	45.0 [40.3, 54.8]	48.0 [44.5, 55.0]	49.0 [45.0, 56.0]	45.0 [40.8, 49.5]	0.747
Total_bilirubin	0.500 [0.400, 0.600]	0.400 [0.375, 0.600]	0.500 [0.400, 0.600]	0.700 [0.500, 0.775]	0.600 [0.525, 0.975]	0.600 [0.500, 0.700]	0.650 [0.400, 0.825]	0.056
Missing	0 (0%)	0 (0%)	0 (0%)	0 (0%)	1 (6.7%)	0 (0%)	0 (0%)	
Direct_bilirubin	0.100 [0.100, 0.100]	0.100 [0.100, 0.100]	0.100 [0.100, 0.100]	0.100 [0.100, 0.175]	0.100 [0.100, 0.175]	0.100 [0.100, 0.100]	0.100 [0.100, 0.200]	0.737
Missing	0 (0%)	0 (0%)	0 (0%)	0 (0%)	1 (6.7%)	0 (0%)	0 (0%)	
Fe+2	76.1 [64.3, 93.0]	67.0 [54.5, 88.4]	77.6 [63.5, 82.5]	74.5 [60.0, 91.8]	81.0 [63.0, 90.0]	77.2 [64.0, 95.0]	87.0 [74.5, 115]	0.713
Missing	0 (0%)	0 (0%)	0 (0%)	0 (0%)	2 (13.3%)	0 (0%)	0 (0%)	
FERRITIN	54.2 [25.9, 96.8]	38.0 [20.5, 53.2]	66.6 [26.6, 124]	59.3 [30.0, 95.3]	37.7 [11.6, 56.1]	44.3 [14.5, 69.2]	54.1 [42.7, 173]	0.290
Missing	0 (0%)	0 (0%)	0 (0%)	0 (0%)	1 (6.7%)	0 (0%)	0 (0%)	
TRANSFERRIN	280 [247, 310]	283 [253, 290]	285 [260, 337]	275 [259, 305]	307 [279, 326]	283 [255, 301]	278 [251, 298]	0.732
Missing	0 (0%)	0 (0%)	0 (0%)	0 (0%)	1 (6.7%)	0 (0%)	0 (0%)	
PLATELETS	256 [213, 290]	245 [228, 251]	249 [220, 308]	267 [208, 324]	242 [212, 278]	243 [212, 260]	255 [227, 298]	0.820
INR	1.00 [1.00, 1.00]	1.00 [0.900, 1.00]	1.00 [0.900, 1.00]	1.00 [1.00, 1.00]	1.00 [1.00, 1.05]	1.00 [1.00, 1.10]	1.00 [0.900, 1.00]	0.063
Missing	0 (0%)	0 (0%)	0 (0%)	0 (0%)	0 (0%)	0 (0%)	1 (8.3%)	
ALBUMIN	4.20 [4.00, 4.33]	4.30 [4.18, 4.50]	4.40 [4.10, 4.58]	4.25 [4.03, 4.40]	4.20 [4.00, 4.45]	4.10 [4.05, 4.45]	4.25 [4.20, 4.50]	0.346
AST_to_ALT	0.850 [0.755, 1.00]	0.670 [0.565, 0.888]	0.920 [0.773, 1.08]	0.795 [0.688, 0.950]	0.875 [0.808, 1.03]	0.840 [0.725, 1.04]	0.880 [0.718, 1.09]	0.631
Missing	0 (0%)	0 (0%)	0 (0%)	0 (0%)	1 (6.7%)	0 (0%)	0 (0%)	
Lymphocytes	2.00 [1.73, 2.58]	2.85 [2.43, 3.03]	1.90 [1.70, 2.50]	1.90 [1.80, 2.90]	1.60 [1.50, 1.90]	2.00 [1.73, 2.28]	1.90 [1.60, 2.40]	0.062
Missing	10 (20.8%)	0 (0%)	9 (40.9%)	3 (16.7%)	2 (13.3%)	1 (5.3%)	3 (25.0%)	
Neutrophils	4.20 [3.53, 4.88]	6.05 [4.85, 7.40]	4.60 [3.50, 5.60]	4.50 [3.45, 6.00]	4.00 [3.40, 4.80]	3.85 [2.88, 4.68]	3.20 [2.90, 3.70]	0.037
Missing	10 (20.8%)	0 (0%)	9 (40.9%)	3 (16.7%)	2 (13.3%)	1 (5.3%)	3 (25.0%)	
Platelets_to_Lymphocytes	126 [98.9, 149]	110 [92.3, 127]	145 [103, 160]	128 [114, 149]	139 [124, 163]	130 [104, 142]	134 [124, 147]	0.374
Missing	10 (20.8%)	0 (0%)	9 (40.9%)	3 (16.7%)	2 (13.3%)	1 (5.3%)	3 (25.0%)	
Neutrophils_to_Lymphocytes	2.00 [1.70, 2.48]	2.20 [1.95, 2.63]	2.10 [1.90, 2.80]	1.80 [1.70, 3.00]	2.00 [1.90, 3.20]	1.90 [1.63, 2.50]	1.70 [1.60, 1.80]	0.158
Missing	10 (20.8%)	0 (0%)	9 (40.9%)	3 (16.7%)	2 (13.3%)	1 (5.3%)	3 (25.0%)	
PNI	53.0 [49.9, 56.9]	55.6 [51.4, 58.6]	51.1 [48.8, 56.0]	52.6 [50.9, 54.5]	51.1 [47.7, 52.9]	51.4 [50.6, 52.1]	52.4 [51.2, 53.5]	0.396
Missing	10 (20.8%)	0 (0%)	9 (40.9%)	3 (16.7%)	2 (13.3%)	1 (5.3%)	3 (25.0%)	
Monocytes	0.50 [0.40, 0.60]	0.75 [0.60, 0.90]	0.50 [0.50, 0.50]	0.50 [0.45, 0.80]	0.40 [0.40, 0.50]	0.50 [0.40, 0.60]	0.50 [0.40, 0.60]	0.016
Missing	10 (20.8%)	0 (0%)	9 (40.9%)	3 (16.7%)	2 (13.3%)	1 (5.3%)	3 (25.0%)	
Lymphocytes_to_Monocytes	4.00 [3.45, 4.80]	3.65 [3.28, 3.98]	4.00 [3.60, 4.30]	3.60 [2.90, 4.95]	4.30 [3.20, 4.90]	4.15 [3.43, 5.40]	3.60 [3.00, 4.70]	0.783
Missing	10 (20.8%)	0 (0%)	9 (40.9%)	3 (16.7%)	2 (13.3%)	1 (5.3%)	3 (25.0%)	
SII	515 [444, 621]	619 [502, 742]	563 [500, 805]	495 [438, 969]	575 [470, 677]	470 [343, 568]	435 [348, 462]	0.165
Missing	10 (20.8%)	0 (0%)	9 (40.9%)	3 (16.7%)	2 (13.3%)	1 (5.3%)	3 (25.0%)	
White_Blood_Cells	8.70 [6.74, 9.78]	11.8 [10.6, 12.7]	9.39 [8.55, 10.5]	10.8 [8.81, 11.4]	6.96 [5.86, 7.60]	6.89 [5.90, 8.80]	6.62 [5.45, 7.58]	0.003
Missing	18 (37.5%)	1 (12.5%)	11 (50.0%)	7 (38.9%)	5 (33.3%)	1 (5.3%)	3 (25.0%)	
C_reactive_protein	7.80 [4.60, 13.9]	4.80 [3.55, 6.80]	6.90 [6.40, 14.9]	5.60 [5.50, 9.20]	7.35 [5.93, 10.2]	8.85 [3.40, 13.4]	4.25 [2.48, 5.50]	0.340
Missing	19 (39.6%)	2 (25.0%)	13 (59.1%)	9 (50.0%)	7 (46.7%)	3 (15.8%)	6 (50.0%)	

Distribution of clinical, metabolic, and inflammatory variables among TDA-defined clusters cl1–cl7. Data are presented as n (%) for categorical variables and median [IQR] for continuous variables. *p*-values indicate overall differences in the seven clusters. Missing denotes the number of participants with unavailable data for the corresponding variable. Abbreviations: noDM, normoglycemia/no diabetes; PreDM, prediabetes; T2DM, type 2 diabetes mellitus; MASLD, metabolic dysfunction-associated steatotic liver disease; MASH, metabolic dysfunction-associated steatohepatitis; BMI, body mass index; AST, aspartate aminotransferase; ALT, alanine aminotransferase; GGT, gamma-glutamyl transferase; HDL, high-density lipoprotein; INR, international normalized ratio; PNI, prognostic nutritional index; SII, systemic immune-inflammation index. Clusters are labelled cl1–cl7, with sample sizes shown in the column headers.

## Data Availability

The affinity proteomics raw data have been deposited to the PRIDE repository with the dataset identifier PAD000040, project DOI: 10.6019/PAD000040.

## Supplementary Materials

The following supporting information can be downloaded at: https://www.mdpi.com/article/10.3390/ijms27094152/s1.

## Abbreviations

The following abbreviations are used in this manuscript: ADA, American Diabetes Association; ALT, alanine aminotransferase; ANOVA, analysis of variance; AST, aspartate aminotransferase; BMI, body mass index; BO, bin overlapping ratio; CASP8, caspase 8; CCL20, C-C motif chemokine ligand 20; CCL28, C-C motif chemokine ligand 28; CD8A, CD8 alpha chain; CDCP1, CUB domain-containing protein 1; CSF-1, colony-stimulating factor 1; CXCL1, C-X-C motif chemokine ligand 1; CXCL6, C-X-C motif chemokine ligand 6; CXCL8, C-X-C motif chemokine ligand 8; CXCL9, C-X-C motif chemokine ligand 9; FDR, false discovery rate; FGF23, fibroblast growth factor 23; FLT3LG, Fms-related tyrosine kinase 3 ligand; FPG, fasting plasma glucose; GGT, gamma-glutamyl transferase; HbA1c, glycated hemoglobin; HGF, hepatocyte growth factor; IQR, interquartile range; IL-6, interleukin 6; IL-8, interleukin 8; IL-10, interleukin 10; IL10RA, interleukin 10 receptor subunit alpha; IL10RB, interleukin 10 receptor subunit beta; K2EDTA, dipotassium ethylenediaminetetraacetic acid; MASLD, metabolic dysfunction-associated steatotic liver disease; NB, number of bins; noDM, normoglycemia; NPX, Normalized Protein eXpression; PCA, principal component analysis; PCR, polymerase chain reaction; PEA, proximity extension assay; PreDM, prediabetes; SLAMF1, signaling lymphocytic activation molecule family member 1; T2DM, type 2 diabetes mellitus; TDA, topological data analysis; VIF, variance inflation factor; WHO, World Health Organization.
