# Supplementary material for: Inflammatory Proteomic Heterogeneity Beyond Glycemia Status in Severe Obesity
Source: Int J Mol Sci. 2026 May 6;27(9):4152. doi: 10.3390/ijms27094152 (PMC13164250; doi:10.3390/ijms27094152)
Supplement: Supplementary file 1 [file ijms-27-04152-s001.zip › ijms-4250566-supplementary/Supplementary_materials_final.pdf]

Inflammatory proteomic heterogeneity beyond glycemic status in severe obesity

Melissa M. Milito [1, 2 (†)], Mattia Chiesa [3 (†)], Alice Mallia [4], Giulia Giusy Papaianni [4], Julia Regalado [1, 2, 5], Claudio Tiribelli [1], Deborah Bonazza [6], Natalia Rosso [1], Silvia Palmisano [1, 7, 8], Cristina Banfi [4 (§), (*)], and Pablo J. Giraudi [1 (§), (*)]

† Equal contribution, § Co-senior authors, equal contribution

Supplementary materials

Table S1 to Table S12 (in the excel file)

Supplementary table legends:

Table S1. Distribution of glycemic status, sex, MASLD stage, and fibrosis in the full bariatric cohort and in the proteomics subcohort.

Data are presented as n (%). The proteomics subcohort was selected from the full bariatric cohort to include all available individuals with prediabetes and to preserve comparable distributions of sex and liver disease severity across glycaemic categories. As a consequence of this design, the proportion of prediabetes differed between cohorts. P-values were calculated using chi-square tests and are provided for descriptive purposes only. MASLD, metabolic dysfunction–associated steatotic liver disease.

Table S2. Full differential expression statistics underlying Figure 1.

Complete differential expression results underlying the volcano plots shown in Figure 1. For each protein, log2 fold change, raw p-values, and Benjamini–Hochberg–adjusted p-values are reported for each glycaemic comparison.

Table S3. Cluster-defining inflammatory proteins identified by topological data analysis

This table summarizes inflammatory proteins contributing to the topological data analysis (TDA)–defined clusters based on plasma proteomic profiles. For each protein, the table reports UniProt ID, gene symbol, protein name, Olink assay identifier, and average NPX expression across the cohort (AveExpr). Log2 fold changes (log2FC) represent the difference in NPX values between each TDA cluster (cl1–cl7) and all remaining samples ("vs. other"). Scaled log2FC values indicate standardized effect sizes across clusters, highlighting proteins selectively enriched or depleted in specific clusters. Positive values indicate higher relative expression within a given cluster, whereas negative values indicate lower expression compared

with the rest of the cohort. P values and Benjamini–Hochberg adjusted p values (adj. P.Val) reflect overall differential expression across clusters.

Table S4-S6. Multivariable regression models using categorical glycaemic status for IL-8, Flt3L and CDCP1 proteins

Multivariable linear regression models examining associations between inflammatory protein NPX levels and categorical glycaemic status (noDM, PreDM, T2DM), adjusted for age, sex, BMI, MASLD stage, antidiabetic therapy, and medication burden score.

Table S7-S9. Multivariable regression models using fasting plasma glucose for IL-8, Flt3L and CDCP1 proteins

Multivariable linear regression models examining associations between inflammatory protein NPX levels and FPG (noDM, PreDM, T2DM), adjusted for age, sex, BMI, MASLD stage, antidiabetic therapy, and medication burden score.

Table S10-S12. Multivariable regression models using glycated haemoglobin for IL-8, Flt3L and CDCP1 proteins

Multivariable linear regression models examining associations between inflammatory protein NPX levels and FPG (noDM, PreDM, T2DM), adjusted for age, sex, BMI, MASLD stage, antidiabetic therapy, and medication burden score.

Supplementary Methods

Figure S1

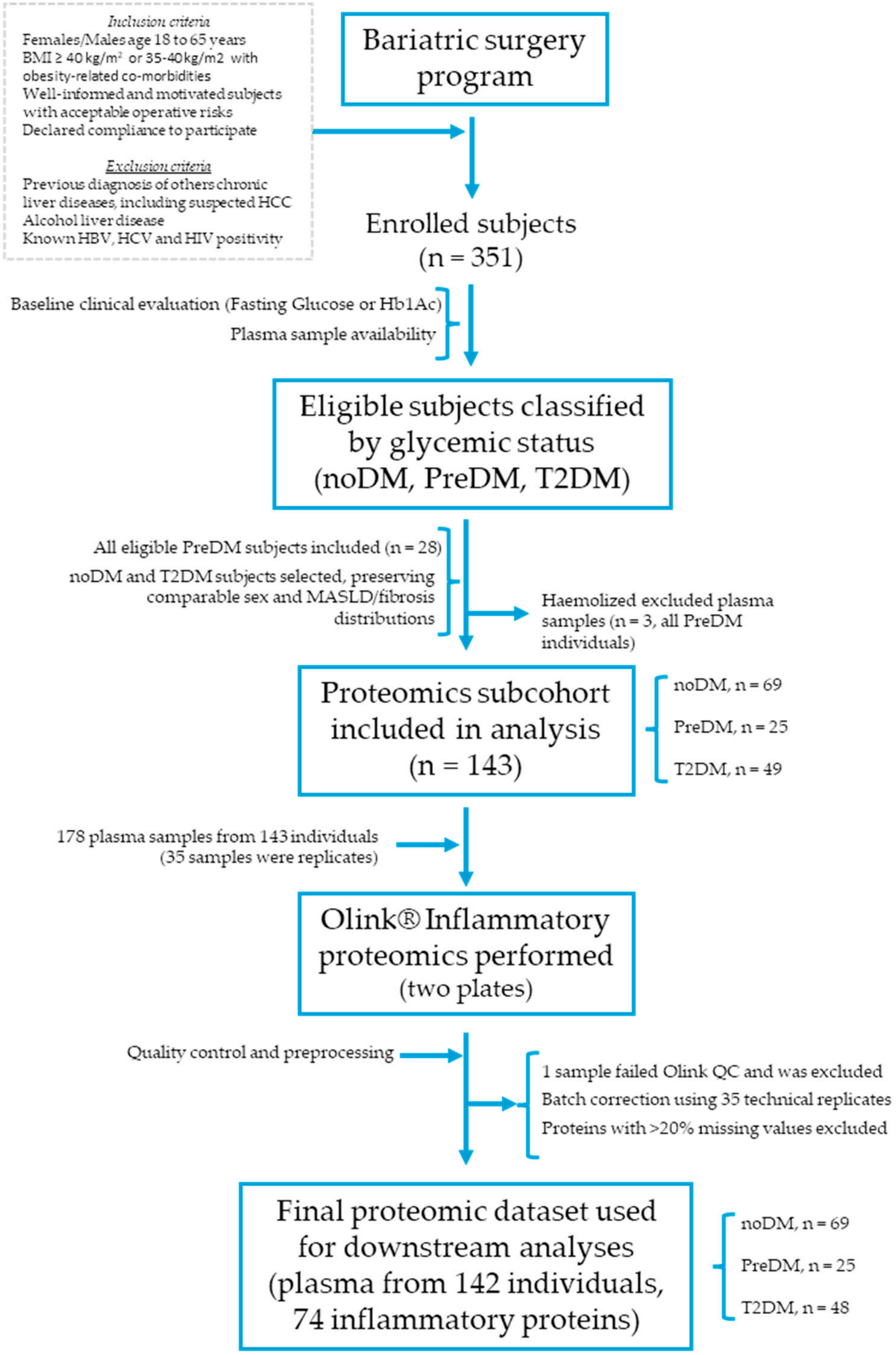

Figure S1. Flow diagram of study design and proteomics subcohort selection

Flow diagram showing selection of the proteomics subcohort from the full bariatric surgery cohort. From 351 enrolled individuals, all available prediabetic participants (n = 28) were initially considered; three were excluded prior to proteomic analysis due to haemolysis, resulting in 25 prediabetic individuals. Normoglycaemic and T2DM participants were subsequently selected to achieve comparable distributions of sex and MASLD/fibrosis severity, yielding a proteomics subcohort of 143 individuals. Plasma inflammatory proteins were measured in 178 samples, including 35 technical replicates used for inter-plate normalization. One sample failed quality control and the corresponding individual was excluded, resulting in a final analytical cohort of 142 individuals used in all downstream analyses.

Batch effect correction and data normalization

Briefly, proteomic data were obtained from two Olink Inflammation panel experiments, generating NPX values for 92 inflammatory proteins measured in a total of 178 plasma samples, 35 of them loaded on both plates. In the pipeline implemented in RStudio using OlinkAnalyze (V.4.3.0), plate identity was explicitly specified, and we performed a two-step correction: (1) bridge normalization using shared samples across both plates with plate 1 as reference and (2) BAMBO normalization using bridge controls. NPX files from the two plates were imported with read_NPX and annotated as plate1 and plate2 before merging for quality control visualization. Bridge normalization was performed with olink_normalization_bridge for inter-plate signal, setting plate1 as reference plate. This bridged matrix was used as the reference for BAMBOO_normalization (LOF threshold = 6). The bridge controls used were the overlapping sample IDs between the reference and sample datasets. Additionally, BAMBOO automatically flagged the outlier samples with low detectability (<6 samples above LOD). Following batch correction, proteins with more than 20% missing NPX values were excluded from further analysis. Outlier samples were identified using principal component analysis, applying a ±3 standard deviation threshold, and removed if they failed quality control. The resulting final dataset comprised 142 samples and 74 inflammatory proteins and was used for all downstream statistical and TDA-based analyses.

Figure S2.

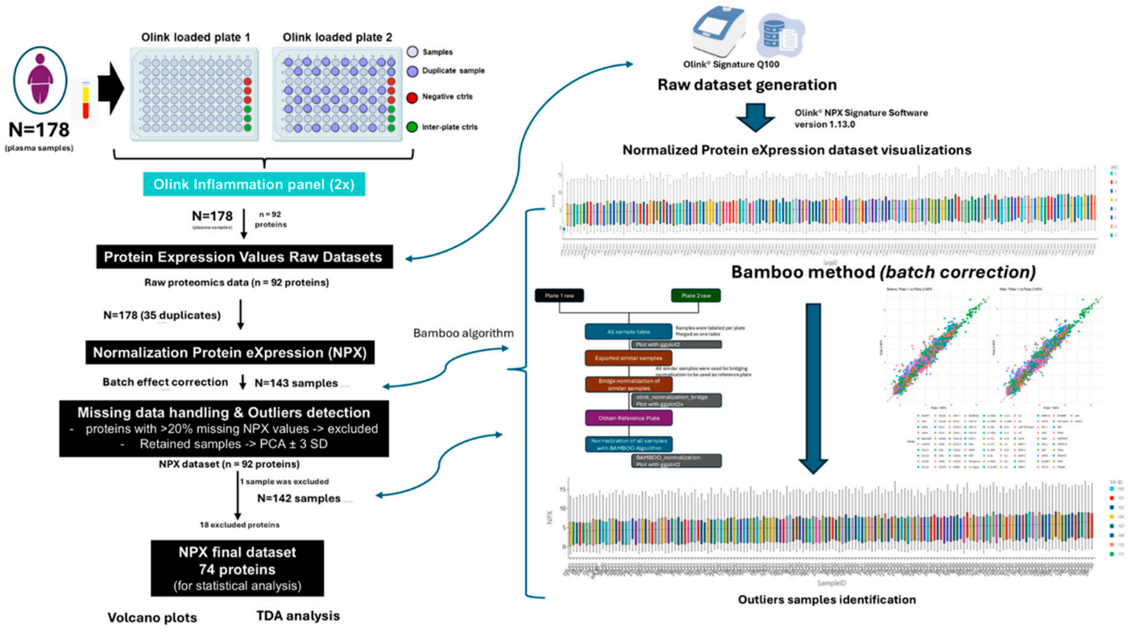

Figure S2. *Plasma inflammatory proteomics workflow and batch effect correction using the BAMBOO method.*

Zoom of Figure S2

Normalized Protein eXpression dataset visualizations (raw data)

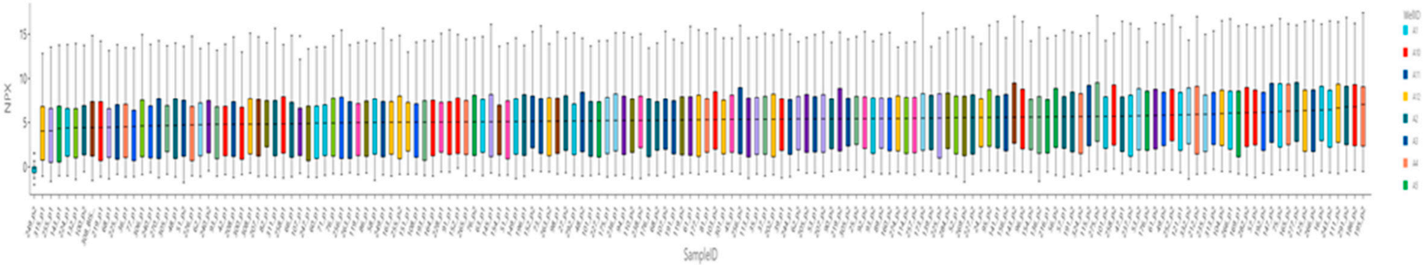

Sample-wise distribution of raw NPX values before batch correction. Each boxplot represents the distribution of Normalized Protein eXpression (NPX) values across the measured inflammatory proteins for each plasma sample in the unprocessed dataset. This visualization was used as an initial quality-control step to assess overall signal distribution, sample-level variability, and potential inter-plate differences prior to normalization and batch correction.

batch correction

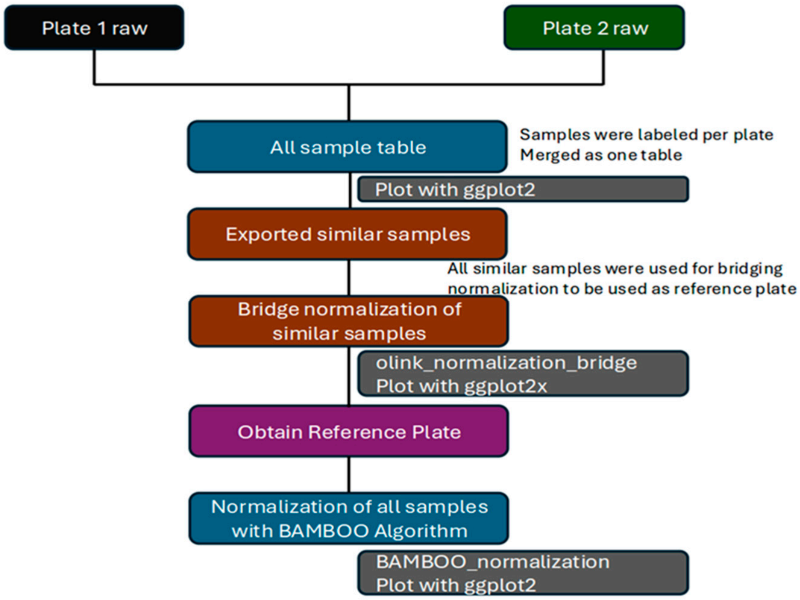

Workflow of data integration, bridge normalization, and BAMBOO-based batch correction. Raw NPX datasets from the two Olink plates were annotated according to plate identity and merged into a single dataset. Shared samples measured on both plates were selected as bridging controls and used for inter-plate bridge normalization with `olink_normalization_bridge`, from which a reference plate was derived. This

reference was then used as input for BAMBOO-based normalization of the full dataset. Plots were generated at each stage to monitor data structure and batch correction performance.

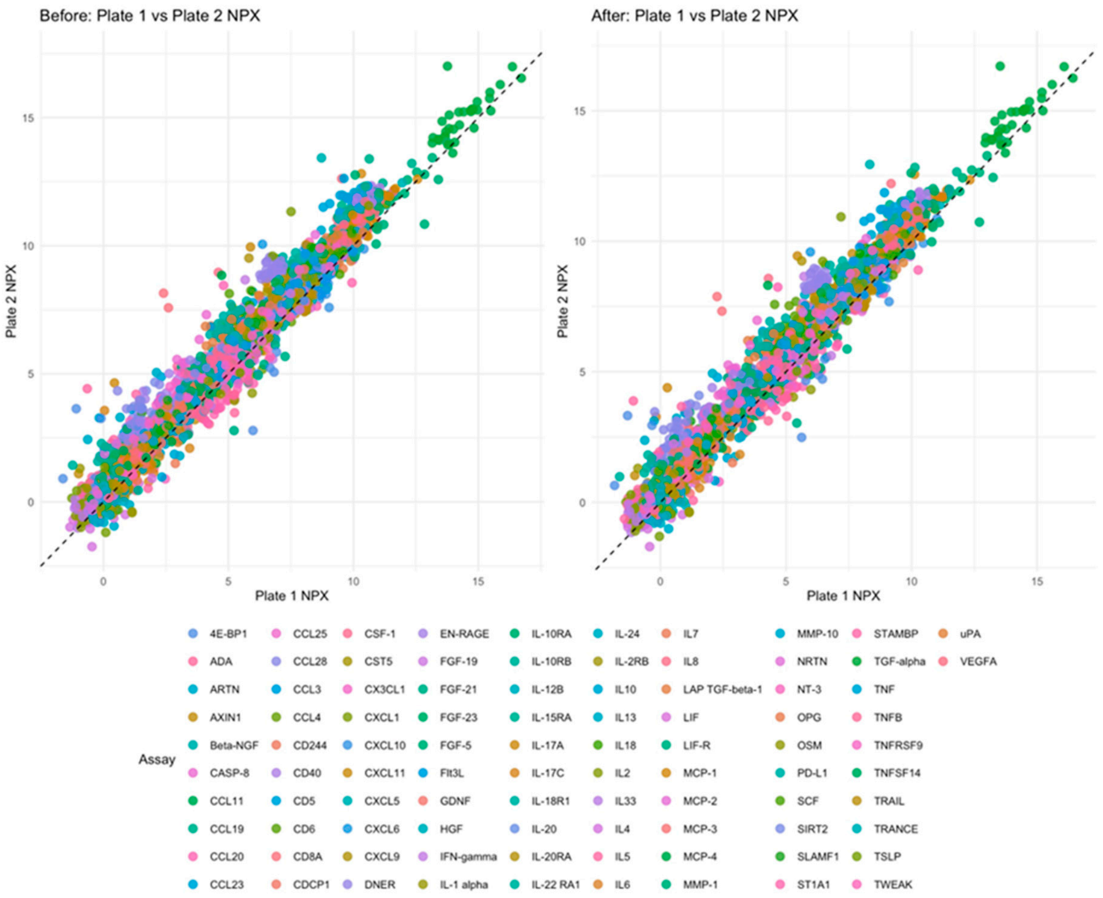

Plate-to-plate agreement of NPX values before and after batch correction. Scatter plots depict NPX values measured in shared bridging samples across plate 1 (x-axis) and plate 2 (y-axis). Each point corresponds to an individual protein measurement. The left panel shows raw NPX values prior to normalization, while the right panel shows values after bridge normalization and BAMBOO correction. The dashed line represents the line of identity (y = x). The increased concordance of points along the identity line after correction indicates improved inter-plate alignment and reduction of batch effects.

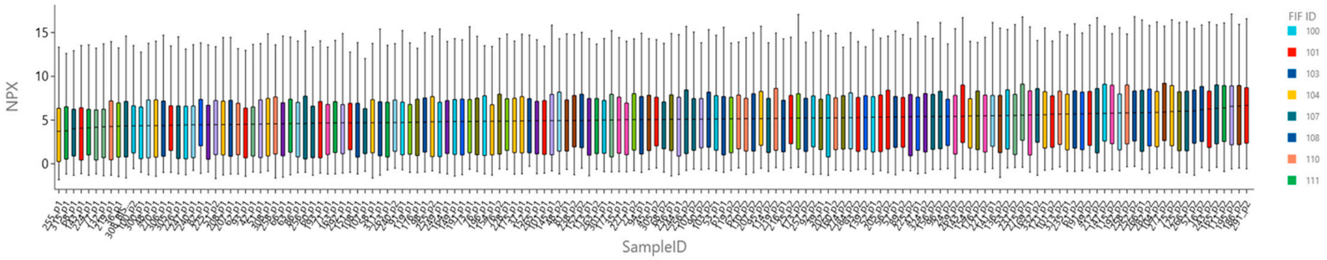

Distribution of NPX values after batch correction and normalization. Boxplots represent the distribution of Normalized Protein eXpression (NPX) values across all measured inflammatory proteins for each sample following bridge normalization and BAMBOO correction. Samples are colored according to plate identity. Compared with the raw data, the more homogeneous distributions across samples indicate effective reduction of inter-plate variability.
